# Adaptive dynamic ϵ-simulated annealing algorithm for tumor immunotherapy

**DOI:** 10.3389/fimmu.2025.1603551

**Published:** 2025-06-18

**Authors:** Xiaoyan Sun, Ying Jiang

**Affiliations:** ^1^ Department of Gynaecology, The People’s Hospital of Liaoning Province, Shenyang, China; ^2^ Department of Gynaecology, Laoning Cancer Hospital and Institute, Shenyang, China

**Keywords:** tumor immunotherapy (TIT), adaptive dynamic ϵ-simulated annealing algorithm (ADϵSA), chemotherapy and immunotherapy, therapeutic strategies, tumor cells

## Abstract

**Introduction:**

Personalized cancer treatment requires precise scheduling of multiple therapeutic agents under biological constraints. Optimizing such regimens is especially challenging due to the nonlinear dynamics of tumor-immune interactions and strict feasibility boundaries. This study aims to develop an intelligent optimization approach capable of handling these complexities within a mathematical tumor treatment model.

**Methods:**

We propose an Adaptive Dynamic ϵ-Simulated Annealing (ADϵSA) algorithm that integrates a multi-population search framework, dynamic ϵ-constraint control, and boundary-aware mutation mechanisms. The algorithm is applied to an improved tumor immunotherapy model (ITIT), formulated using ordinary differential equations (ODEs) based on established experimental and clinical studies. The model incorporates tumor cells, immune effector cells, and three types of anti-tumor drugs: chemotherapy, immunotherapy, and anti-angiogenic agents.

**Results:**

Simulation experiments were conducted on twelve classical benchmark functions to evaluate the convergence performance and robustness of the algorithm. ADϵSA demonstrated strong global search capability, fast convergence, and solution stability. When applied to the ITIT model, the algorithm successfully identified optimal drug dosing schedules that significantly reduced simulated tumor burden—from ~1500 to below 500 cells—while maintaining treatment within physiologically acceptable limits.

**Discussion:**

Unlike traditional metaheuristics such as PSO or GA, which are less suited for constraint-rich, dynamic ODE-based systems, ADϵSA offers structural advantages in trajectory feasibility and adaptive convergence. This study highlights the potential of biologically informed optimization algorithms in personalized oncology and provides a computational basis for future closed-loop, patient-specific treatment strategies.

## 1 Introduction

Currently, common clinical tumor immunotherapies include monoclonal antibody therapy, immune checkpoint blocker therapy, pericyte therapy, lytic virus therapy, and tumor vaccine (1). Among them, dual immunotherapy is a novel tumor immunotherapy approach, and its 4-year follow-up results show a 3-year OS rate of 25%, which is a onefold improvement compared with chemotherapy (2). In addition, the mechanism and progress of tumor immunotherapy combined with radiotherapy and targeted therapy are also in progress. Chemotherapeutic agents act synergistically in immunotherapy through multiple mechanisms, such as immunogenic cell death (ICD) (3, 4).

Immunotherapy is a treatment method that uses the body’s own immune system to recognize and attack tumor cells. It mainly uses immunotherapeutic drugs to activate or enhance the function of immune cells so that they can overcome the escape and resistance of tumor cells (5). Chemotherapy is a treatment method that uses chemical drugs to kill or inhibit tumor cells, and it is mainly administered intravenously or orally to attack tumor cells throughout the body without discrimination. The advantages and disadvantages of immunotherapy and chemotherapy are also different (6, 7). The advantages of immunotherapy are that it has relatively few side effects and does not cause much damage to normal cells, and once it works, it can last for a long time and may even lead to long-term survival or cure. However, the disadvantage is that it is slow to work, it requires waiting for the immune system to respond and adjust, and not all patients benefit from it, requiring biomarker testing to predict which patients are suitable (8). The advantages of chemotherapy are that it works faster, controls tumor growth, and spreads rapidly and is suitable for many types and stages of tumors, especially for tumors with high sensitivity. However, the disadvantage is that the side effects are relatively large and can cause some toxicity and damage to normal cells, leading to adverse reactions such as hair loss, vomiting, anemia, and infection, and over time, tumor cells may develop drug resistance, making chemotherapy less effective (9–11).

Intelligent optimization algorithm is a method that uses artificial intelligence techniques to find the optimal solution (12). It can be applied to several aspects in the field of oncology therapy, such as drug discovery, drug combination, dose adjustment, and personalized therapy. The development of intelligent optimization algorithms in the field of oncology therapy is an emerging field that can help physicians better understand the evolutionary dynamics of tumors and thus better formulate treatment plans (13). Over the past decade, specific mathematical frameworks to quantify the evolutionary dynamics of cancer have been the basis for revolutionizing precision oncology, promising to map the past and future of individual cancers (14). The application of intelligent optimization algorithms in the field of oncology therapy has been achieved primarily by modeling the evolutionary dynamics of tumor progression, treatment resistance, and metastasis. The field also involves multiple aspects of high-throughput data analysis, genomics, computer science, and mathematical modeling (15, 16).

There have been several experimental and clinical studies of intelligent optimization algorithms in oncology therapy, with some progress and results (17)—for example, an intelligent optimization algorithm based on reinforcement learning (GENTRL) can design a new antitumor compound in 21 days compared to a traditional timeline of approximately 1 year (18). A second-order algebraic algorithm-based intelligent optimization platform (QPOP) can significantly reduce the number of experiments and data required to identify drugs and doses for optimal combination therapy design and has discovered some unexpectedly effective drug combinations (19). A neural-network-based intelligent optimization platform (CURATE.AI) can adjust multidrug doses using only patient data, dynamically linking dose to optimal tumor reduction and safety and significantly improving treatment outcomes without the need for large data and complex genetic information (20). A game-theoretic-based intelligent optimization algorithm (AT-1) can iteratively reduce the dosage of antitumor drugs, prevent drug-resistant cells from outnumbering drug-sensitive cells after tumor shrinkage is observed, and improve tumor control and survival (21).

Simulated annealing algorithm is a flexible and efficient global optimization algorithm with significant advantages in solving complex optimization problems (22–24). Its core idea is derived from the solid annealing process, which finds the optimal solution of the problem through the dynamic balance between global search and local search. In the field of oncology treatment, simulated annealing algorithms are widely used in problems such as personalized treatment optimization, drug dosage design, and radiotherapy schedule optimization (25). Tumor treatment usually relies on a combination of chemotherapeutic drugs and immune drugs, but there are significant differences in drug tolerance and efficacy among different patients (26, 27). Drug dosage design is a complex optimization problem that requires balancing the therapeutic effect with side effects. SA can dynamically adjust the dosage ratio of immune drugs to chemotherapeutic drugs to optimize the treatment plan. In the early stage of treatment, a higher dosage is provided to rapidly inhibit the growth of tumor cells, and in the middle and late stages, the dosage is gradually reduced to minimize the toxic side effects. Personalized drug dosage regimens are designed for individual patient differentiation to enhance therapeutic efficacy (28, 29). Model parameters were sourced from previously published immunotherapy models (25–28), which are based on preclinical data and human-scale estimations. These parameters reflect biologically realistic rates for tumor growth, immune stimulation, and drug clearance. Tumor types, stages of disease progression, and immune system status vary widely among patients, and treatment plans need to fully take into account individualized needs. SA can also dynamically optimize treatment plans by combining the patients’ genetic information and tumor characteristics, synergistically optimizing the combination strategy of multiple treatments (30). Considering the patients’ dynamic response to treatment, the cycles of chemotherapy and immunotherapy can be dynamically optimized through adaptive adjustment of the optimized treatment process, balancing the intensity of treatment and patient tolerance (31). A multi-stage treatment strategy can also be designed to flexibly adjust the treatment intervals according to the treatment effect and tumor response (32).

In this paper, we design a metaheuristic algorithm with constraints in continuous space, adaptive dynamic ϵ-Simulated annealing algorithm (ADϵSA). It has a population intelligence with multiple groups, and the algorithm’s strategy of finding the best can be changed dynamically according to the dimensionality of the problem and the variables of constraints. In solving the problem of oncology treatment, the weight of immunotherapy and chemotherapy drugs can be intelligently assigned. The improved tumor immunotherapy (ITIT) model is also updated and modified to add an endothelial cell model and an anti-angiogenic drug concentration model. The tumor–immune treatment interaction model used in this study is based on clinically informed differential equations adapted from published human and murine studies. No physical cell lines or animal subjects were used; all simulations are *in silico* based on established biological dynamics.

The rest of the paper is outlined as follows: The improved TIT model is proposed in Section II. The ADϵSA algorithm is proposed in Section III. Section IV validates the effectiveness and feasibility of the algorithm by testing on popular benchmarking sets. Section V applies ADϵSA to ITIT for medical strategy formulation, which makes the drug minimize the harm to the human body.

## 2 Improved tumor immunotherapy

This part adds the endothelial cell influence to the original TIT model. The effect of anti-angiogenic drugs is also added to generate the ITIT model, which can be a richer and more accurate mathematical model to describe tumor growth. In the following model, E(t) denotes the number of endothelial cells. *T*(*t*) represents the number of tumor cells, *I*(*t*) represents the number of immune cells, *Con_che_* (*t*) represents the blood concentration of chemotherapy drugs, *Con_im_* (*t*) represents the blood concentration of immunotherapy drugs, and *Con_ant_* (*t*) represents the blood concentration of anti-angiogenic drugs.

Chemotherapeutic agents can interfere with DNA replication or division of tumor cells, which can lead to apoptosis or necrosis. These methods can reduce the number and activity of tumor cells, thus inhibiting tumor growth and metastasis. Immune checkpoint inhibitors can release the tumor’s suppression of the immune system and enhance the recognition and killing of tumor cells by T cells, thus increasing the clearance of tumor cells by immune cells. These methods can also reduce the number and activity of tumor cells, thereby inhibiting tumor growth and metastasis. The growth model of tumor cells is shown below:


(1) $$\begin{array}{c} T(t+1)=T(t)+\vartheta_{1}\times T(t)\times \left(1-\vartheta_{2}\times T(t)\right)\\ -\gamma \times T(t)\times I(t)-\epsilon \times T(t)\times Con_{che}(t) \end{array}$$

where $\vartheta_{1}$ denotes the rate of self-growth of immune cells, $\vartheta_{2}$ denotes the rate of influence between immune cells and tumor cells, γ denotes the rate of growth of tumor cells when they are attacked, and ϵ denotes the stress coefficient of tumor cells to chemotherapeutic agents. Equation 1 includes additive tumor reduction from both immune-mediated cytolysis and chemotherapy-driven apoptosis.

The role of immune cells in tumor therapy may be dual, inhibiting tumor growth and metastasis as well as promoting tumor escape and progression. Chemotherapeutic agents can kill rapidly dividing tumor cells, but they also damage normal hematopoietic stem cells and immune cells, thereby reducing the number and activity of immune cells. Immune checkpoint inhibitors can release the tumor’s suppression of the immune system and enhance the activity and persistence of T cells, thus increasing the number and function of immune cells.


(2) $$\begin{array}{c} I(t+1)=I(t)+\vartheta_{3}-\lambda \times I(t)\\ +\frac{\alpha_{1}\times T^{2}(t)\times I(t)}{\beta_{1}+T^{2}(t)}+\frac{\alpha_{2}\times T(t)\times Con_{im}(t)}{\beta_{2}+Con_{im}(t)}\\ -\xi_{1}\times T(t)\times I(t)-\xi_{2}\times Con_{che}(t)\times I(t) \end{array}$$

where $\vartheta_{3}$ is the rate of immune cell autopoiesis, λ is the rate of natural attenuation of immune cells, $\alpha_{1}$ indicates the maximum rate of immune cell aggregation, $\alpha_{2}$ indicates the maximum rate of impact of immunotherapy on tumor cells, $\beta_{1}$ indicates the rate of decline brought about by tumor cells, $\beta_{2}$ indicates the rate of decline induced by immunotherapy, $\xi_{1}$ indicates the rate of immune cell stress to chemotherapeutic agents, and $\xi_{2}$ indicates the rate of tumor cell stress to immune cells. Equation 2 models immune cell growth driven by tumor-antigen presence and boosted by immunotherapy, whereas chemotherapy induces moderate immunosuppression via a linear decay term.

Tumor cells and endothelial cells suppress the immune system in a number of ways, thereby reducing the number of immune cells. Chemotherapy drugs kill cancer cells, but they also kill normal rapidly dividing cells, including immune cells, thus reducing the number of immune cells. Immunotherapy increases the number of immune cells by activating or enhancing the body’s own immune system to kill cancer cells. See Equation 3 for details.


(3) $$\begin{array}{c} A(t+1)=A(t)+\vartheta_{3}+\eta \times T(t)\\ +\xi_{3}\times A^{2}(t)\times I(t)-\frac{\alpha_{3}\times A(t)\times Con_{ant}(t)}{\beta_{3}+A^{2}(t)} \end{array}$$

In general, chemotherapeutic drugs are cytotoxic; they kill rapidly dividing tumor cells and also affect the growth and division of normal cells. The concentration of chemotherapeutic drugs in the blood varies with the time of administration and the rate of metabolism, usually peaking sometime after administration and then gradually declining. See Equation 4 for details.


(4) $$Con_{che}(t+1)=\chi_{che}(t)-e^{-\theta_{1}}Con_{che}(t)$$

where $\chi_{che}(t)$ represents the concentration of chemotherapeutic drugs. $\theta_{1}$ is the attenuation rates of chemotherapy drugs.

Immune drugs are a class of medications that activate or suppress the immune system, and they help the body recognize and destroy tumor cells. The concentration of immune drugs in the blood also varies with the time of administration and the rate of metabolism but is generally more persistent than chemotherapy drugs, with some immune drugs remaining in the blood for weeks or even months. See Equation 5 for details.


(5) $$Con_{im}(t+1)=\chi_{im}(t)-e^{-\theta_{2}}Con_{im}(t)$$

where $\chi_{im}(t)$ represents the concentration of immune drugs. $\theta_{2}$ is the attenuation rates of immune drugs.

Anti-angiogenic drugs are a class of drugs that can block the formation of blood vessels in tumors. They can cut off the nutrient supply to tumors, thus inhibiting tumor growth and metastasis. The concentration of anti-angiogenic drugs in the blood also varies with the time of administration and the rate of metabolism, but they are generally more stable than chemotherapeutic drugs, and some anti-angiogenic drugs can remain in the blood for days or even weeks. See Equation 6 for details.


(6) $$Con_{ant}(t+1)=\chi_{ant}(t)-e^{-\theta_{3}}Con_{ant}(t)$$

where $\chi_{ant}(t)$ represents the concentration of anti-angiogenic drugs. $\theta_{3}$ is the attenuation rates of anti-angiogenic drugs.

Chemotherapeutic drugs refer to the application of chemically synthesized drugs for the treatment of tumors, and their principle of action is to inhibit the growth and metastasis of tumors by killing fast-growing cells, including cancer cells and normal cells. Immunological drugs refer to targeting some specific proteins or signaling pathways on the surface of tumor cells to achieve the purpose of tumor treatment by activating or inhibiting the immune function of the body. Anti-angiogenic drugs refer to targeting the process of tumor angiogenesis through inhibiting the proliferation, migration, and differentiation of vascular endothelial cells, thus cutting off the nutrient supply and oxygen delivery of the tumor to achieve the purpose of tumor treatment. Combining the above information, we can get the integrated objective function. From formulas (1)–(6), we can get Equation 7:


(7) $$\begin{array}{c} F_{min}=\sum_{t=t_{0}}^{t}\delta^{t}\{\omega \times T^{2}(t)+\int_{0}^{\chi_{che}(t)}tan^{-1}\left({\bar{U}}_{1}^{-1}\times s\right)\times {\bar{U}}_{1}\times R_{1}\times ds\\ \begin{array}{l} +\int_{0}^{\chi_{im}(t)}tan^{-1}\left({\bar{U}}_{2}^{-1}\times s\right)\times {\bar{U}}_{2}\times R_{2}\times ds\\ +\int_{0}^{\chi_{ant}(t)}tan^{-1}\left({\bar{U}}_{3}^{-1}\times s\right)\times {\bar{U}}_{3}\times R_{3}\times ds \end{array}\} \end{array}$$

where ${\bar{U}}_{1}$ and ${\bar{U}}_{2}$ respectively represent the maximum allowable dose of chemotherapy drugs and the dose of a single injection of immunizing agent, δ is the discount factor, and ω is a constant coefficient.

## 3 Adaptive dynamic ϵ-simulated annealing algorithm

For real-world constrained optimization problems, the expression without loss of generality is shown below. See Equation 8 for details.


(8) $$\begin{array}{l} \;\text{Minimize},\;f(x),\;x=\left(x_{1},\;x_{2},\;\cdots ,\;x_{n}\right)\\ \text{S}\text{u}\text{b}\text{j}\text{e}\text{c}\text{t}\;\text{t}\text{o}:\;g_{i}(x)\leq 0,\;i=1,\;2,\;\cdots ,\;p\\ \;h_{j}(x)=0,\;j=p+1,\;p+2,\;\cdots ,\;m \end{array}$$

where *f*(*x*) denotes the objective function, *n* denotes the dimension of the variable, *p* denotes the number of inequality constraints, *m* denotes the number of equality constraints, and *g*(*x*) and *h*(*x*) denote equality and inequality constraint.

Average constraint violation is the extent to which constraints are violated during the optimization process. The smaller the average constraint violation is, the closer the solution of the algorithm is to the feasible domain. In practice, we usually want the solution of the algorithm to satisfy all of the constraints, so we need to minimize the average constraint violation as much as possible. The global overall average constraint violation is shown below. See Equation 9 for details.


(9) $$MCV=\frac{\sum_{i=1}^{p}\max\left(g_{i}(x),0\right)+\sum_{j=p+1}^{m}\max\left(\left|h_{j}(x)\right|-\mu ,0\right)}{m}$$

where *μ* is a small tolerance that is equal to 1E-4.

The dynamic constraint factor ϵ means that in the optimization process, the value of the constraint factor ϵ gradually decreases with the increase of the number of iterations, which makes the constraints gradually become stricter. This has the advantage of ensuring the convergence and stability of the algorithm as well as improving the solution efficiency of the algorithm. If the value of the constraint factor ϵ is set too small, it may cause the algorithm to converge too slowly; if it is set too large, it may cause the algorithm to fail to converge. The dynamic constraint factor ϵ is used to dynamically adjust the weight of the constraints according to the current optimization conditions in order to better control the search direction and speed of the algorithm. It can help the algorithm to converge to the optimal solution faster and can avoid the algorithm from falling into the local optimal solution. See Equation 10 for details.


(10) $$\epsilon =\left\{ \begin{array}{l} \epsilon_{\max}\times \exp\left(-s\times \frac{t}{t_{\max}}\right),t\leq 0.6t_{\max}\\ 0,\;t>0.6t_{\max} \end{array}\right.$$

where *t* represents the number of current evolutionary iterations, and *t*_max_ represents the total number of evolutionary iterations. *ϵ*_max_ represents the maximum term of constraint violation in the primitive population.

Boundary constraint processing technology refers to the optimization process, the value range of the variables to limit, so that the optimization process will not exceed the predetermined range of variables. In practice, many problems need to limit the range of variables, such as temperature and pressure, in the production process. We propose the adaptive boundary constraint processing technique, which is a processing method for constrained optimization problems. It can adaptively adjust the boundary constraints according to the changes in the optimization process so as to ensure the rationality and feasibility of the optimization results. The flow chart of the specific algorithm is shown below. See Algorithm 1 for details.

Algorithm 1 **ADBCHT scheme.**


1 **for** i = 1 to N **do**
2  **for** j = 1 to N **do**
3   
$x_{i,l}=x(<lowerbound)$
;
4   
$x_{i,u}=x(>upperbound)$
;
5   
$x_{j,l}=x(==-\inf)$
;
6   
$x_{j,u}=x(==\inf)$
;
7   
r=randn(1,1)
;
8   
$\begin{array}{c} r_{l}=rand\times abs(lowerbound)/\\ \left(\left(lowerbound-x_{i,l})+abs(lowerbound\right)\right) \end{array}$
;
9   
$\begin{array}{c} r_{u}=rand\times abs(upperbound)/\\ \left(\left(x_{i,u}-upperbound)+abs(upperbound\right)\right) \end{array}$
;
10   
$x_{i,l}=(1-r_{l})\times lowerbound+r_{l}\times x_{i,l}$
;
11   
$x_{i,u}=(1-r_{u})\times upperbound+r_{u}\times x_{i,u}$
;
12   
$x_{j,l}=r\times lowerbound$
;
13   
$x_{j,u}=r\times upperbound$
;
14  **end for**

15 **end for**

The final improved simulated annealing algorithm combines the novel constraint handling above to form the adaptive dynamic ϵ-simulated annealing algorithm. The specific pseudo-code is as follows. See Algorithm 2 for details.

Algorithm 2 **Adaptive dynamic ϵ-simulated annealing algorithm.**


1   Initialization parameters
2     Set initial solution *x*
_current_

3     Initial temperature *T* = *T*
_0_

4     Minimum temperature Tmin
5     Cooling factor alpha ∈ (0, 1)
6     Maximum number of iterations maxiter
7   Define the objective function *f*(*x*) and the constraint *g*(*x*).
8   Define the penalty function *P*(*x*) which is used to constrain the penalty term for violation.
9   Set current best solution *x*
_best_ = *x*
_current_

10   Enter the outer loop (temperature loop)
11   while *T* > *T*
_min_

12     for *i* = 1 to *max*
_iter_

13      Generate new solution *x*
_new_

14      Generate a random candidate solution *x*
_new_ near the current solution *x*
_current_.
15     Check if *x*
_new_ satisfies constraint *g*(*x*
_new_)
16     If not, calculate penalty term
17      *f*
_new_ = *f*(x_new_) + *P*(x_new_)
18      Calculate the target difference ΔE
19      ΔE = *f*
_new_ - *f*(*x*
_current_)
20      Accept the criterion
21     if ΔE< 0
22      *x*
_current_ = *x*
_new_

23     if *f*new< *f*(*x*
_best_)
24      *x*
_best_ = *x*
_new_

25     else
26      prob = exp (-ΔE/T)
27     if random (0, 1)< prob
28      *x*
_current_ = *x*
_new_

29     Cooling
30     *T* = alpha * *T*

31     Output the optimal solution
32   return *x*
_best_, *f*(*x*
_best_)

## 4 Simulation and analysis for ADϵSA

To validate the performance of our algorithm in complex optimization problems, we design a series of benchmark tests based on 12 popular test functions for experimental simulation. We employ ADϵSA and conduct multiple independent tests for each benchmark function to ensure the robustness of the results. The search range of each function is determined based on its definition domain to ensure coverage of the global optimal point and its surrounding region. The maximum number of iterations is 100. The initial temperature is set to 1000°C to simulate annealing with gradual cooling. The temperature decay coefficient is set to 0.95, and the temperature decreases nonlinearly with iterations. The acceptance criterion is based on the change of the objective function value, and the probabilistic acceptance mechanism is adopted to balance the global and local search. Through the optimization process of the 12 test functions above, we aim to verify the global search capability of the algorithm and whether it can effectively jump out of the local optimum and locate the global optimum point. The algorithm demonstrates high convergence efficiency and achieves rapid optimization within a limited number of iterations. It also exhibits strong adaptability to a wide range of function types.

Although widely used in general-purpose optimization, algorithms such as PSO and GA are not inherently suited to constraint-driven, dynamic ODE-based problems such as the one addressed here. ADϵSA, with its built-in ϵ-constraint scheduling, simulated annealing convergence control, and boundary-aware design is structurally more appropriate for solving tumor immunotherapy planning problems embedded in nonlinear dynamical systems. Future work may explore modified PSO/GA versions with constraint-handling layers, but such additions would fundamentally alter their core simplicity.

**Figure 1:**
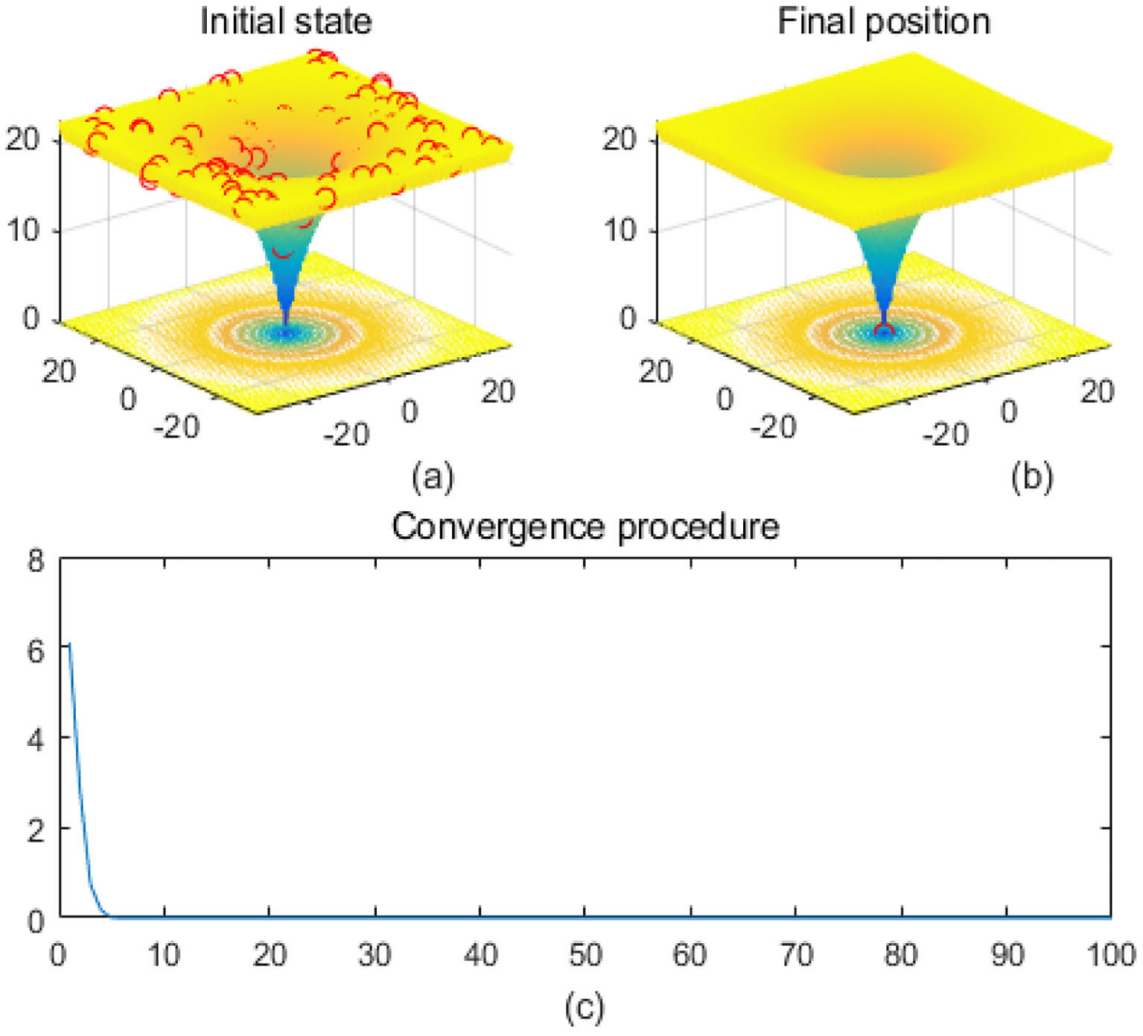
Ackley. **(a)** is the initial state diagram, **(b)** is the final state diagram, and **(c)** is the convergence curve diagram.

**Figure 2:**
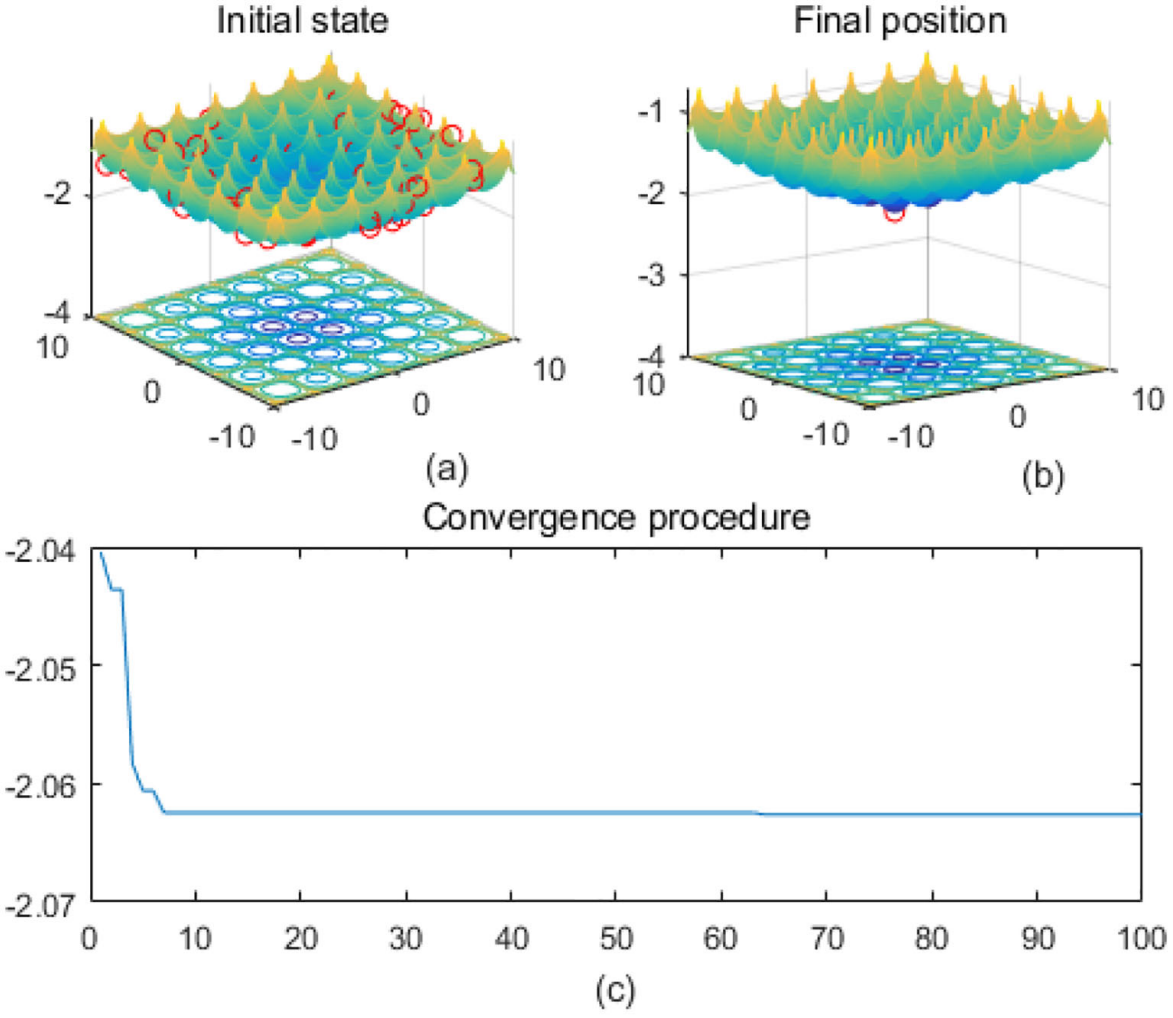
Cross-in-tray. **(a)** is the initial state diagram, **(b)** is the final state diagram, and **(c)** is the convergence curve diagram.

**Figure 3:**
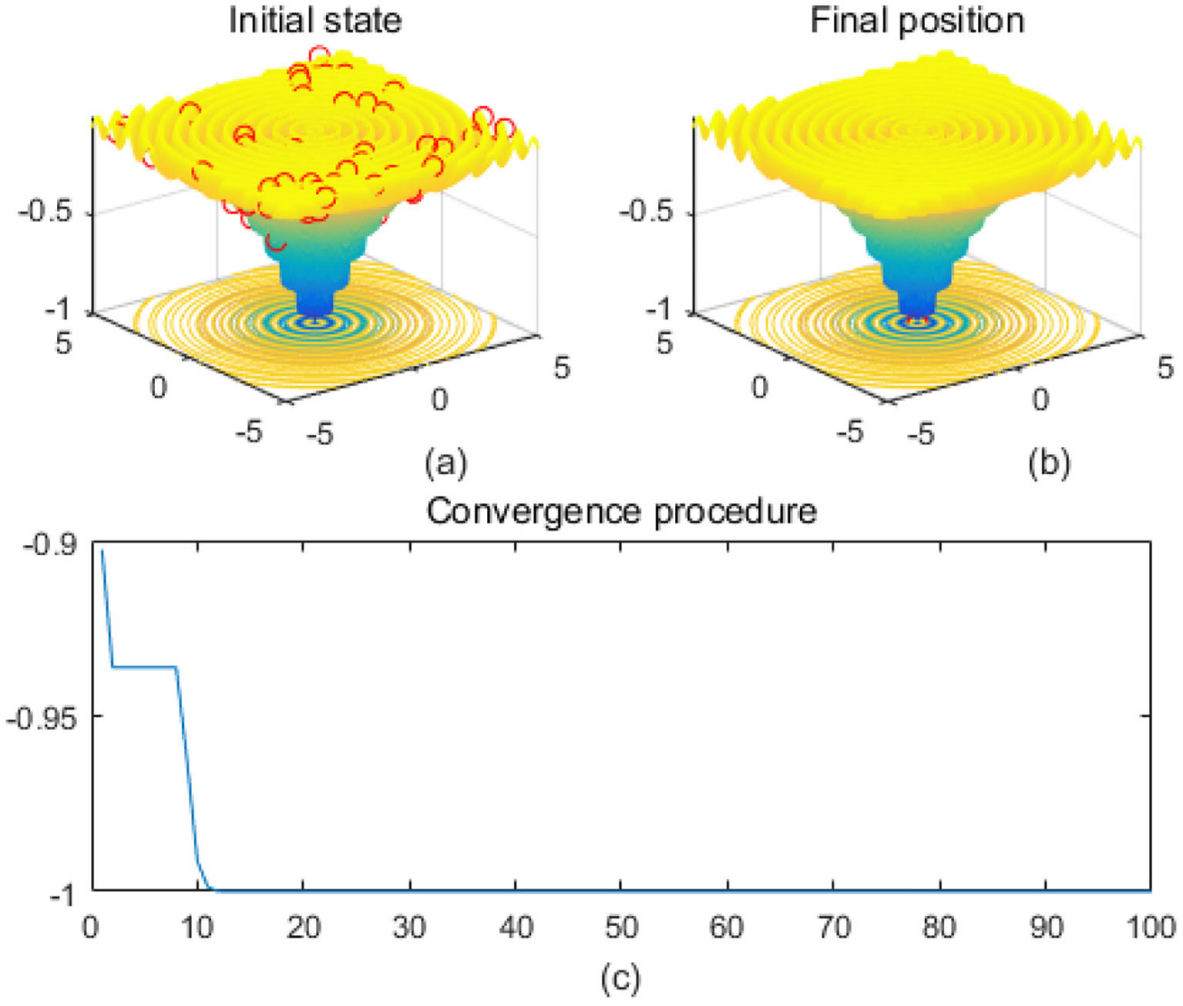
Drop-wave. **(a)** is the initial state diagram, **(b)** is the final state diagram, and **(c)** is the convergence curve diagram.

**Figure 4:**
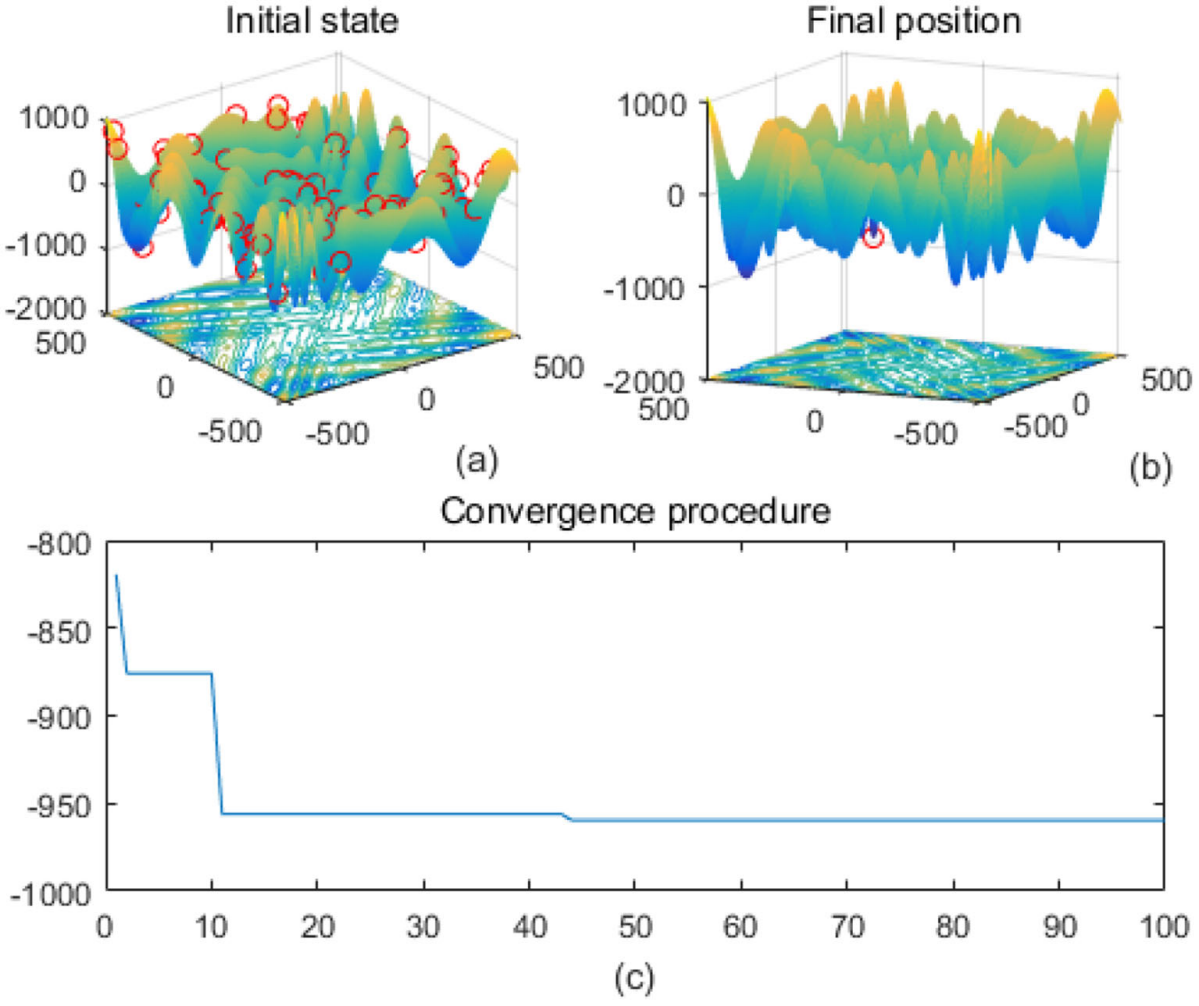
Eggholder. **(a)** is the initial state diagram, **(b)** is the final state diagram, and **(c)** is the convergence curve diagram.

**Figure 5:**
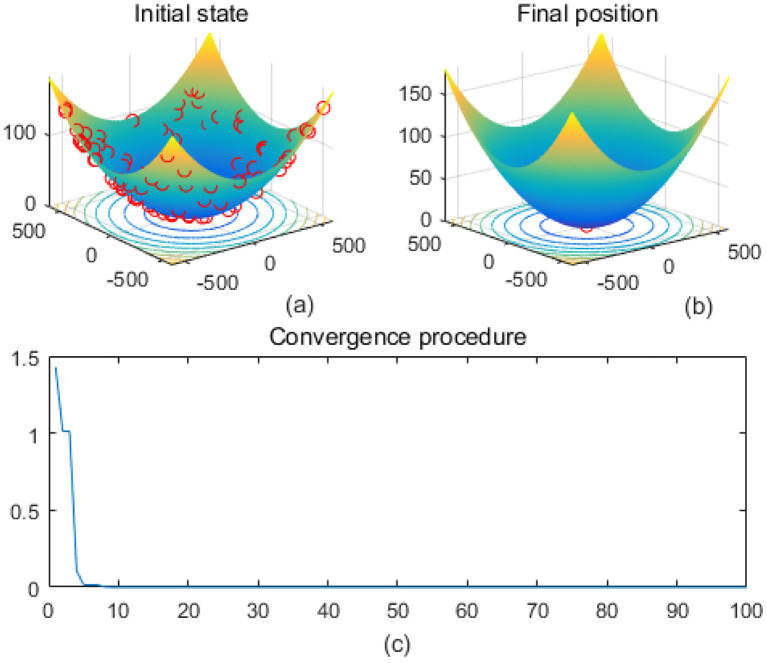
Griewank. **(a)** is the initial state diagram, **(b)** is the final state diagram, and **(c)** is the convergence curve diagram.

**Figure 6:**
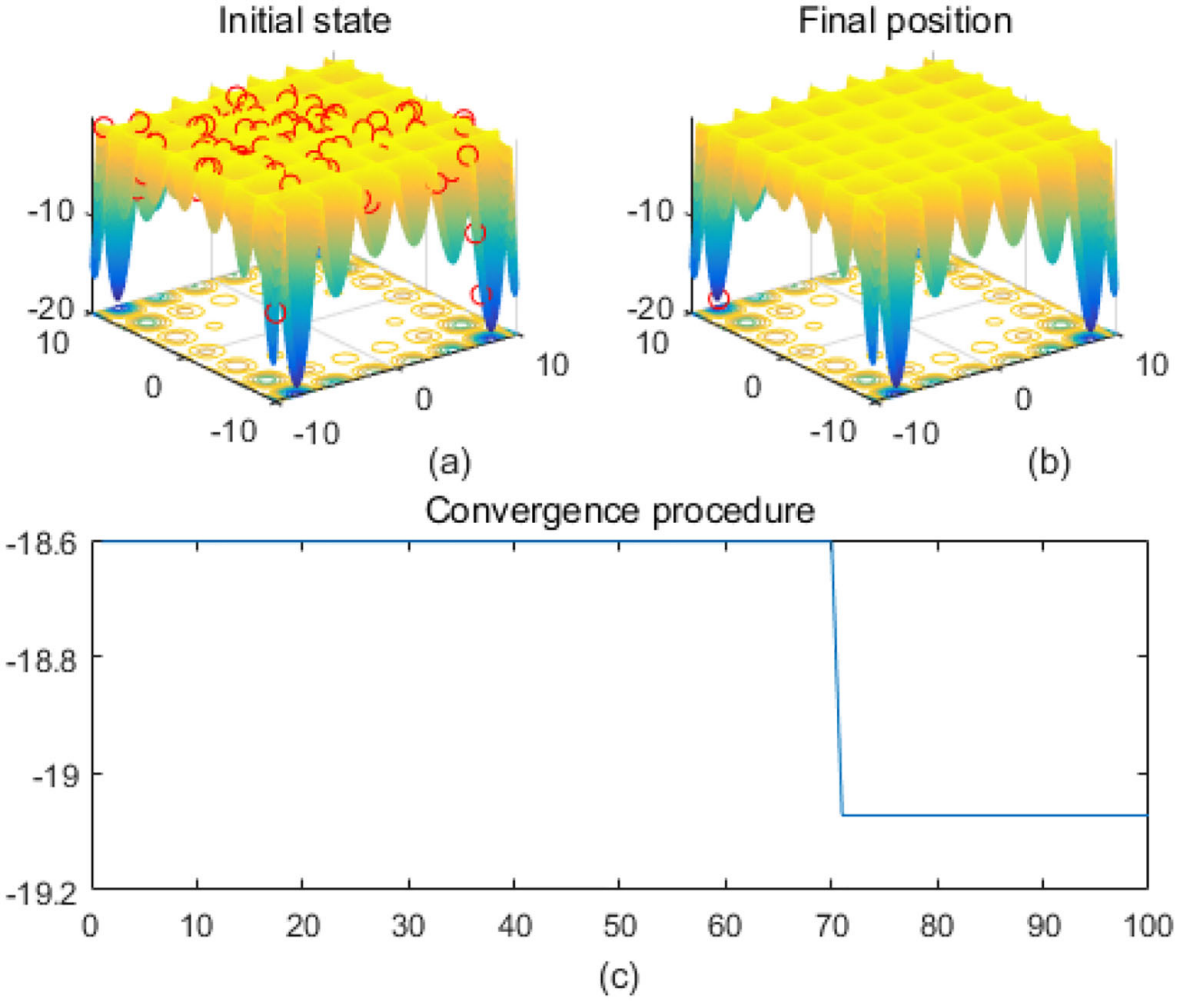
Holder Table. **(a)** is the initial state diagram, **(b)** is the final state diagram, and **(c)** is the convergence curve diagram.

**Figure 7:**
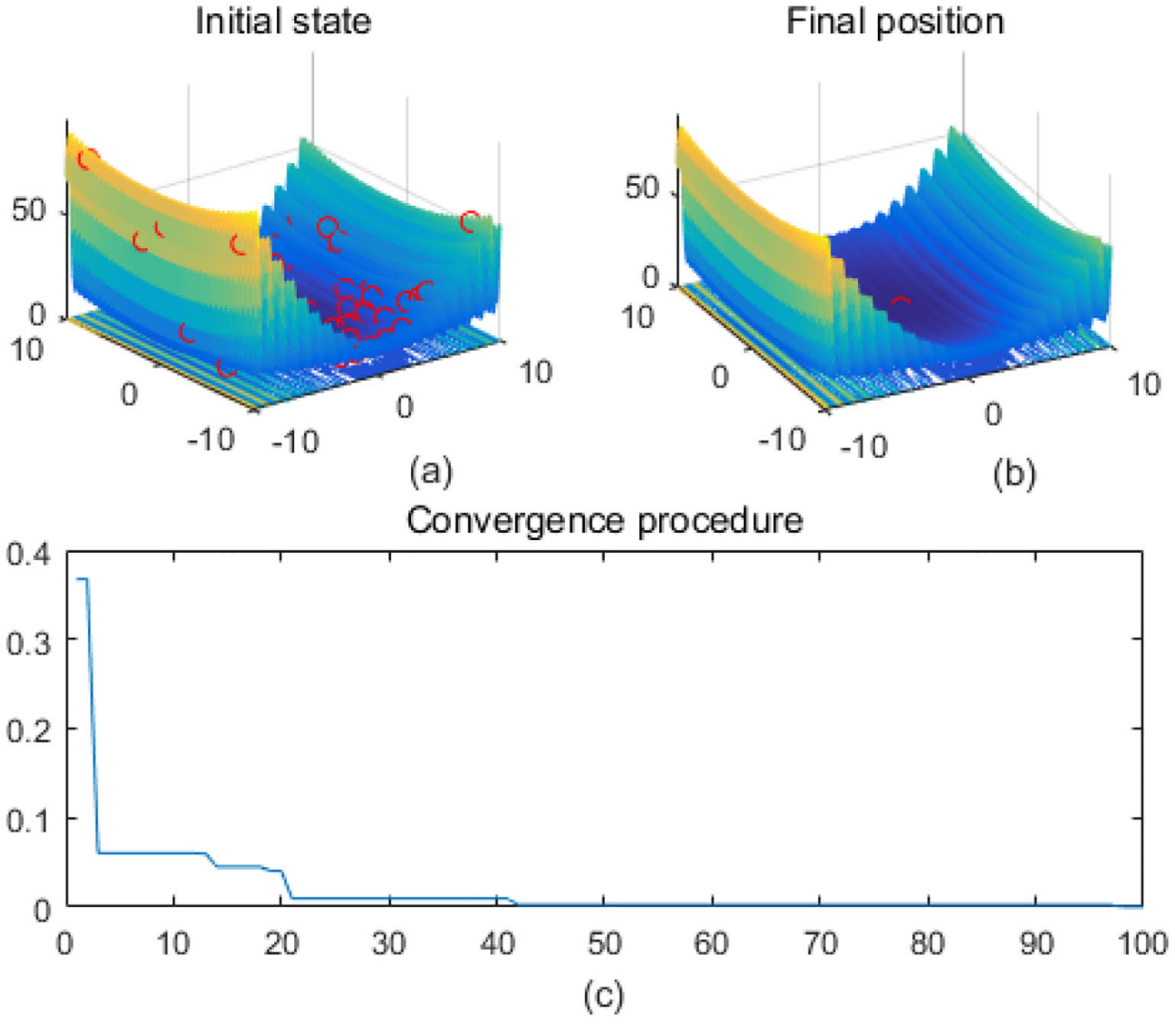
Levy. **(a)** is the initial state diagram, **(b)** is the final state diagram, and **(c)** is the convergence curve diagram.

**Figure 8:**
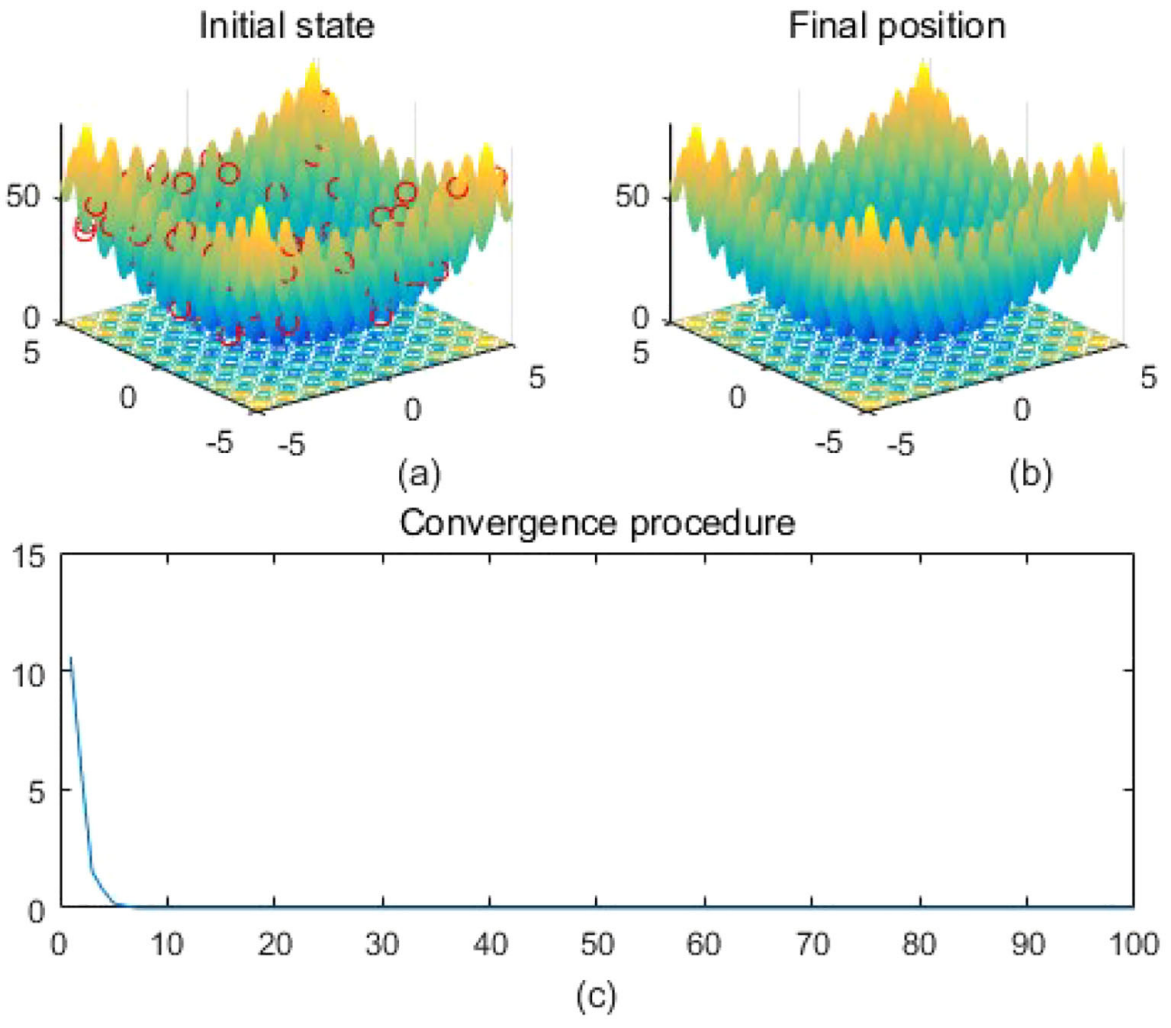
Rastrigin. **(a)** is the initial state diagram, **(b)** is the final state diagram, and **(c)** is the convergence curve diagram.

**Figure 9:**
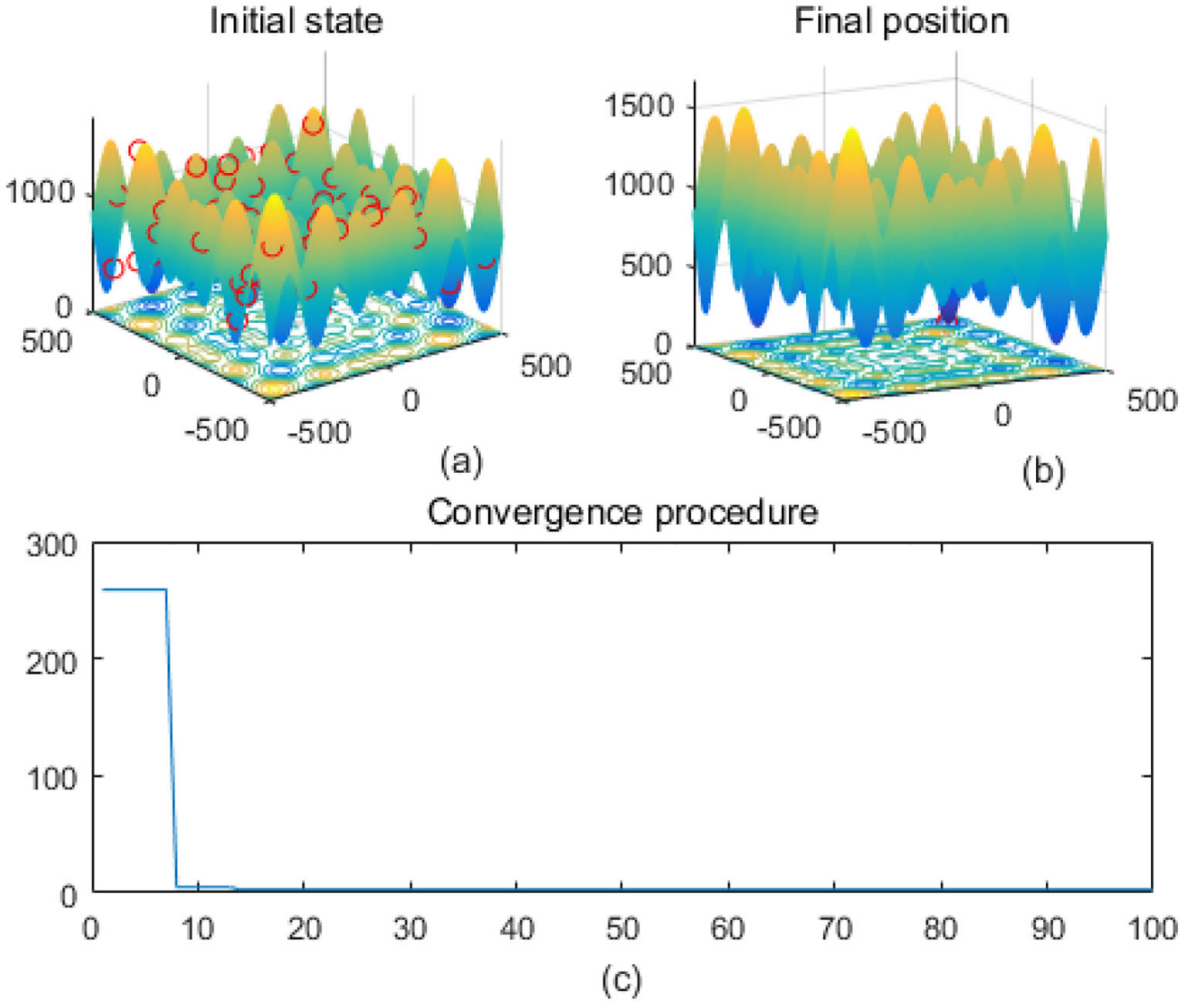
Schwefel. **(a)** is the initial state diagram, **(b)** is the final state diagram, and **(c)** is the convergence curve diagram.

**Figure 10:**
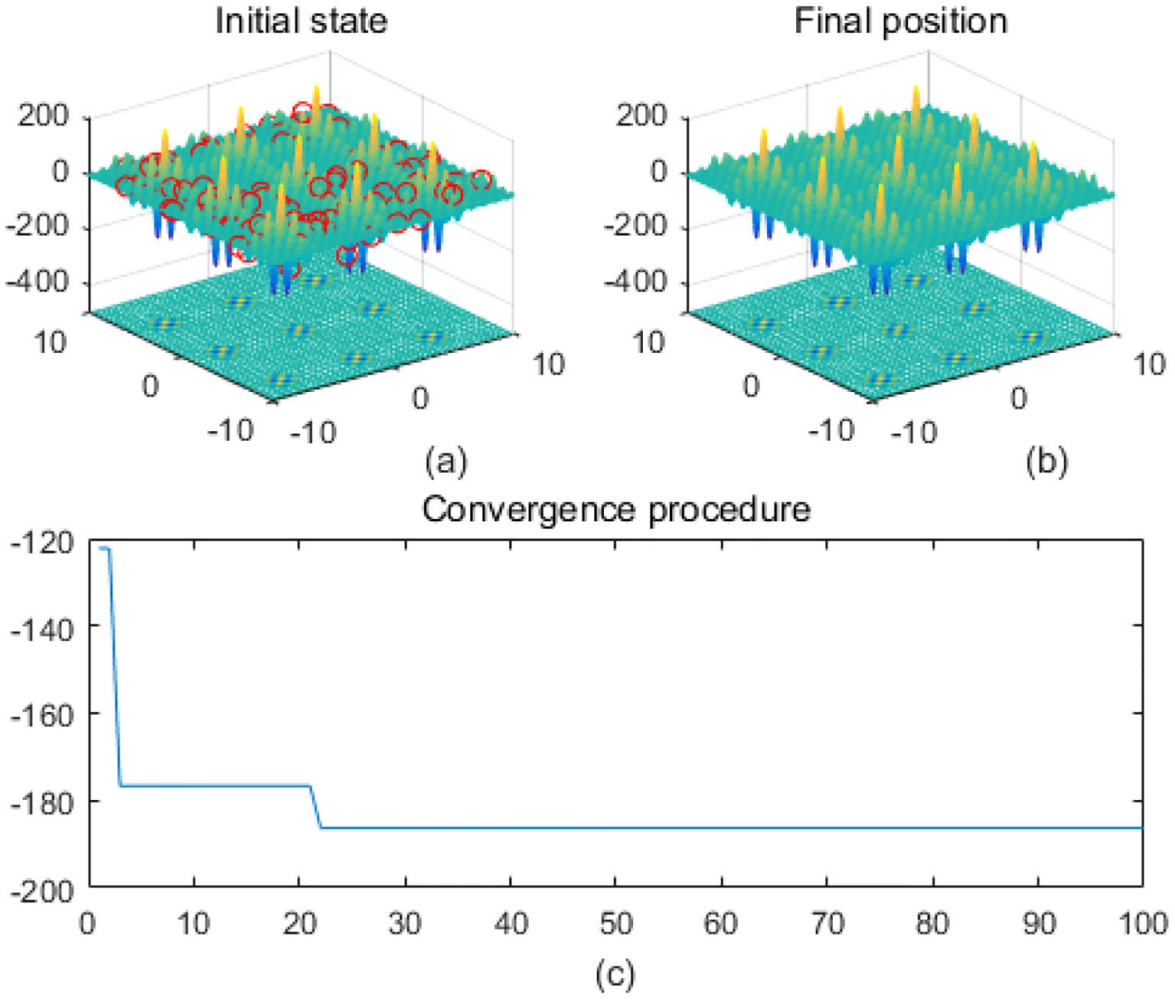
Shubert. **(a)** is the initial state diagram, **(b)** is the final state diagram, and **(c)** is the convergence curve diagram.

**Figure 11:**
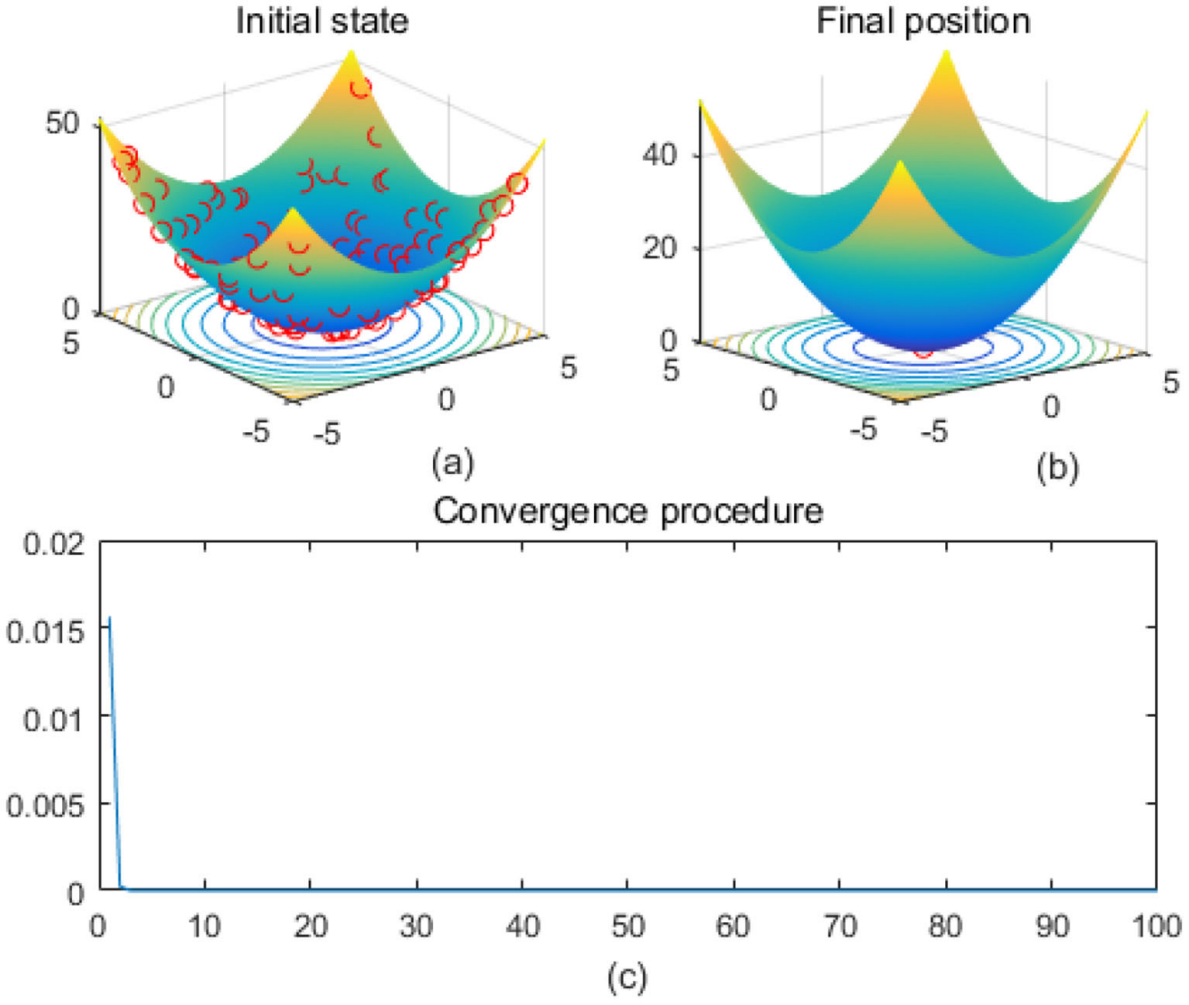
Sphere. **(a)** is the initial state diagram, **(b)** is the final state diagram, and **(c)** is the convergence curve diagram.

**Figure 12:**
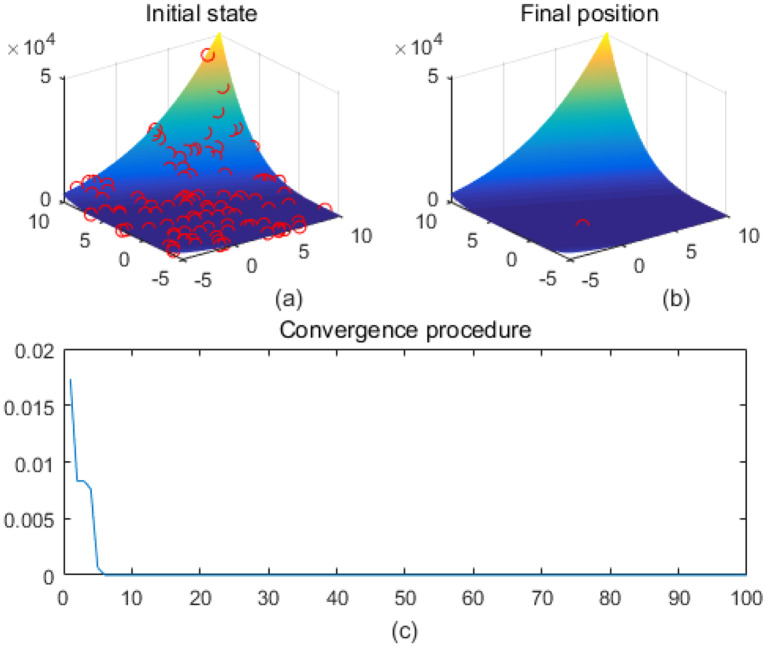
Zakharov. **(a)** is the initial state diagram, **(b)** is the final state diagram, and **(c)** is the convergence curve diagram.

Note that the scaling of axes in **Figures 1**–**12** is adapted to each function’s numerical characteristics and standard domain definitions. These differences reflect the inherent heterogeneity in function value ranges and convergence behavior, which are essential to preserve when analyzing algorithmic adaptability and performance across multimodal vs. unimodal, low-range vs. high-range objective spaces. Axis scaling is function-specific to preserve the numerical resolution and convergence trajectory appropriate to each benchmark function’s characteristic output range.

The overall performance of the algorithm on different test functions is shown. These test functions cover a variety of challenging optimization scenarios such as single-peak, complex multi-peak, periodic, and concave–convex mixing. The algorithm performs well in global search on single-peak and partially multi-peak functions, which can quickly converge to the global optimal solution with smooth convergence curves, such as Sphere, Zakharov, Griewank, etc. The algorithm also performs well on functions with complex multi-peak structures, such as Sphere, Zakharov, and Griewank. On the functions with complex multi-peak structure, the algorithm shows strong local search ability and can find lower fitness regions, such as the Eggholder and Shubert functions, but there is still the phenomenon of falling into the local optimum in some cases. The fitness curves show that the algorithm shows fast convergence on most of the tested functions, usually reaching the optimal or near-optimal solution within the first 20 iterations, with good stability in the later stages. For simple structures and low-complexity functions, the algorithm results are highly stable, and the solutions are concentrated in the global optimum; in high-complexity functions, there is a scattering of individual solutions.

**Figure 1a** presents the initial state, and the 3D surface plot shows the initial population distribution. From the figure, it can be seen that the population is scattered throughout the search space of the Ackley function. The red points represent the initial individuals, which are distributed at different locations on the function surface. The Ackley function has multiple local minima, but the global minimum is at the center (dark blue region at the bottom of the function). **Figure 1b** presents the final position, and the 3D surface plot shows where the individuals of the population have finally converged after the optimization process. It can be seen that the individuals have mostly converged to the global optimal point of the Ackley function. The optimization algorithm successfully finds the global minimum of the solution space. **Figure 1** presents the convergence process of (c), with the convergence graph showing the convergence speed of the optimization algorithm. The horizontal axis is the number of iterations, and the vertical axis is the objective function value. The curve shows that the function value decreases rapidly at the initial iterations, indicating that the algorithm finds a better solution quickly in the early stages. As the number of iterations increases, the curve flattens out, indicating that the algorithm converges to the global optimum point. **Figure 2** shows the surface of the cross-in-tray function, characterized by multiple cross-wave peaks and local minima. The global minimum is about -2.062 and is distributed in several symmetric locations. The fitness curve shows that the algorithm quickly finds the optimum after the first few iterations. The curve is smooth with no significant fluctuations, indicating that the algorithm is stable and effective.

Multi-peak functions with multiple local optima are used to test the ability of an algorithm to jump out of a local optimum. **Figure 3** shows the drop-wave function surface, characterized by a ripple-like multi-peak structure with a distinct global minimum in the center. The line graph shows that the fitness value decreases rapidly and stabilizes within 10 iterations, approaching the optimal value of -1. The function surface has periodic ripples with multiple local minima, increasing the search difficulty. The smoothness of the fitness curve indicates that the solution converges stably and without oscillations. **Figure 4** shows the Eggholder function surface, characterized by high complexity, multiple peaks and undulations, difficult search space, and multiple local minima. The solution distribution in the right panel converges to a low fitness region but is not fully concentrated, indicating that the algorithm finds a near-optimal solution but may not reach the global optimum point. The lower line graph shows a gradual decrease in the fitness values, with a fast initial convergence followed by a stabilization and limited local search. It is completely focused to the global optimum point and may be plagued by local minima. The algorithm performs better initially but gets stuck in a local search bottleneck later and fails to fully optimize the complex surface. Effectiveness can be improved by introducing stronger global search strategies.

**Figure 5** shows the Griewank function surface, with an overall bowl-like structure but with many small fluctuating local minima. The fitness curve shows that the algorithm converges to the optimum in very few iterations. After convergence, the curve is smooth with no significant fluctuations. **Figure 6** shows the Holder Table function surface, showing a complex multi-peak structure in the form of a grid with deep local minima at the bottom. **Figure 6b** shows that the solution is successfully concentrated at the bottom of some global optimum point, indicating that the algorithm searches the optimal region efficiently. The fitness curve shows that the algorithm stagnates in the early stage, quickly jumps out of the local trap after about 50 iterations, and finally converges to the global optimum value -19.2085 The initial search stays in the local region, then breaks through the local optimum point in the late stage, and reaches the global optimum quickly. The fast convergence at the later stage indicates that the algorithm has a mechanism to jump out of the local trap.

Extremely complex structured functions with sharp fluctuations and irregular structure are used to test the performance of algorithms on complex functions. **Figure 7** shows the multi-peak Levy function surface. The function overall shows multiple fluctuating valley structures. The fitness curve shows that the algorithm converges quickly in the first 20 iterations and then remains stable, indicating that the algorithm finds the optimal solution efficiently. **Figure 8** shows that on the surface of the Rastrigin function, the function has a periodic multi-peak structure, with obvious fluctuations, and the global minimum is at (0,0). The solution is highly concentrated in the global optimum as shown in **Figure 8b**, which indicates that the algorithm effectively jumps out of the local minima and finds the global optimal solution successfully. **Figure 9** shows the Schwefel function surface. The function has a complex multi-peak structure with significant fluctuations, and the global minimum is hidden in a deeper location, which poses a challenge to the optimization process. **Figure 9b** shows that the optimization algorithm converges to a low fitness region, but the solution is not completely concentrated near the global minimum, which suggests that the algorithm has a local convergence phenomenon on the surface of the complex function. The fitness curve shows that the algorithm rapidly reduces the fitness value in the first 20 iterations, and then it tends to stabilize, but finally it does not reach the theoretical optimal value of f(x) = 0.

Highly fluctuating and multi-peaked function surfaces contain high-frequency fluctuations and multiple local minima to test the algorithm’s ability to adapt to small-scale variations. **Figure 10** shows the Shubert function surface. The function is characterized by a high-density multi-peak structure, and there are a lot of local extreme points, which make optimization more difficult. The fitness curve shows that the algorithm rapidly reduces the fitness value and then converges stably in the lower fitness region, without continuing to decline significantly. **Figure 11** shows the sphere function surface of the function. The function has a standard bowl-like structure with the minimum value at the center (0,0). The fitness curve shows that the algorithm converges to the optimal value quickly in the first few iterations and then stays smooth, showing efficient convergence performance. **Figure 12** shows the surface of the Zakharov function, the overall bowl structure. The distribution of the solutions is scattered. The center of the function is the global minimum. The fitness curve shows that the algorithm quickly converges to the optimal value in the first 20 iterations, and there is almost no change in the subsequent iterations, which shows the fast and stable convergence ability.

Although no explicit sensitivity sweeps of ϵ_max_ or α are included, their effects can be observed from convergence behaviors across multiple benchmark functions. The consistent success of the algorithm on both simple (e.g., sphere) and complex (e.g., Rastrigin, Eggholder) functions suggests that the chosen parameter configuration (ϵ_max_ = 100, α = 0.95) strikes an effective balance between early exploration and late-stage convergence. This is evident from the delayed but ultimately accurate descent seen in rugged functions and rapid convergence in smooth, convex problems.

In summary, ADϵSA converges quickly, with most functions converging within the first 10–20 iterations. The performance is stable, and the algorithm performs accurately and centrally for single-peaked functions and some multi-peaked functions. It demonstrates strong global search ability on some complex functions (e.g., Rastrigin and Holder Table). However, on complex multi-peak functions (e.g., Eggholder and Shubert), the algorithm tends to fall into local optimality and fails to find the global optimal solution completely. For highly complex search spaces, some of the solutions are not sufficiently centralized, and the search accuracy and global search strategy need to be improved.

## 5 Develop therapeutic strategies for tumor immunotherapy using ADϵSA

In this section, we apply the ADϵSA algorithm proposed in Section III to the TIT model proposed in Section II as an experimental verification. According to clinical treatment ITIT, chemotherapeutic drugs and immune drugs are used as input, and the cost of treatment loss is used as the objective function. Through the iteration of the ADϵSA algorithm, the optimal therapeutic strategies for patients with a certain basic condition are worked out. According to clinical medical statistics borrowed, the specific parameters of the dynamic models are presented in **Table 1**.

**Table 1:** Experimental parameter.

Parameters	Estimated value	Units
*ϑ*_1_	0.00431	*day* ^-1^
*ϑ*_2_	1.02×10^-9^	*cell* ^-1^
*γ*	6.41×10^-11^	*cell* ^-1^
*ε*	0.08	*day* ^-1^
*λ*	0.204	*day* ^-1^
*ξ*_1_	3.42×10^-6^	*cell^-1^*
*ξ*_2_	2×10^-11^	*day* ^-1^
*α*_1_	0.0125	*day* ^-1^
*α*_2_	0.125	*day* ^-1^
*β*_1_	2.02×10^7^	*cell* ^2^
*β*_2_	2×10^7^	*cell*
*θ*_1_	0.1	*day* ^-1^
*θ*_2_	1	*day* ^-1^
*δ*	0.95	*N/A*
*ω*	0.1392×10^-4^	*N/A*

Based on the details above, we have completed the establishment of the TIT model and determined the specific value of the cost function according to clinical ITIT. At the same time, the feasibility and effectiveness of the ADϵSA algorithm are also verified on benchmarks. ADϵSA was applied to the model of TIT to develop therapeutic strategies. The best processing strategy is obtained through experiments, which proves the effectiveness and feasibility of the algorithm. The cost function is designed to minimize the number of tumor cells and also to use the smallest dose of chemotherapeutic drugs and immune drugs to achieve the least harm to the human body.

**Figure 13:**
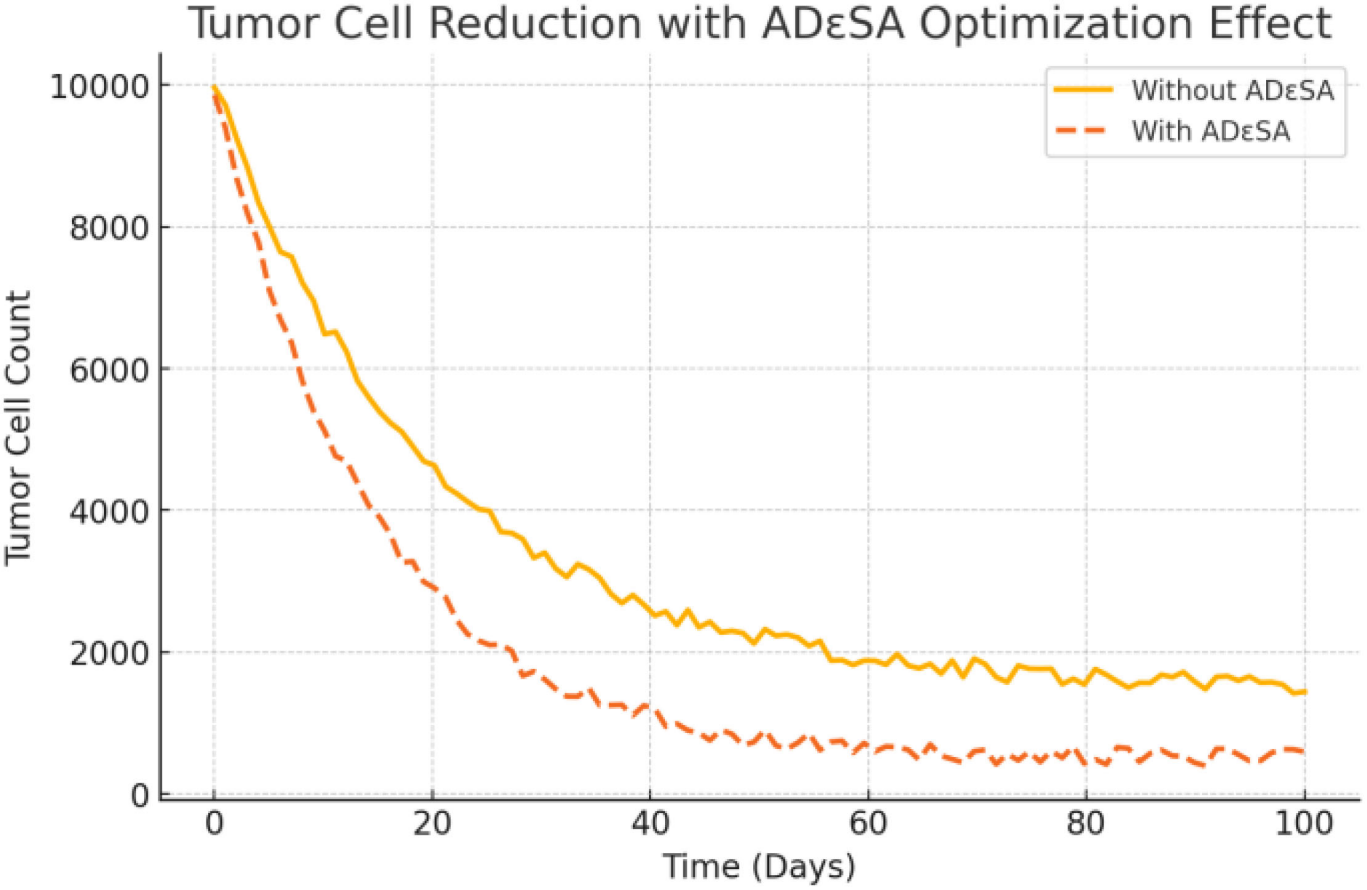
Curve of the number of tumor cells.

**Figure 13** shows the simulation results of the tumor cell number over time, comparing the effect in two cases, using the ADϵSA method and not using the method. The following scientific analysis in terms of tumor cell dynamics, optimization capability, and algorithm performance fully demonstrates the superiority of the ADϵSA method. The yellow solid line represents the cell decline curve without using the ADϵSA method. In the initial stage, the number of tumor cells decreases slowly, indicating that the optimization speed is low. It eventually stabilizes at a higher level (~1,500), indicating that the treatment without the optimized method only inhibits tumor growth to a limited extent. Higher fluctuations in cell counts may indicate that the model is not robust enough and sensitive to parameter perturbations. The orange dashed line represents the cell decline curve after using the ADϵSA method. The initial decline is significantly accelerated, and the number of tumor cells decreases rapidly, indicating that the ADϵSA method can find the optimal treatment strategy more efficiently. Eventually, it stabilizes at around 500, which is significantly lower than the convergence value of the unused method, reflecting the stronger global search capability of the ADϵSA method. The fluctuation amplitude is significantly reduced, indicating that ADϵSA improves the robustness and stability of the system. The curve with ADϵSA significantly accelerates the decline in the early stage and quickly finds a better solution. This fast convergence property is attributed to the effective balance between global search and local exploitation by ADϵSA, which avoids the search stagnation of traditional methods in complex optimization problems.

**Figure 14:**
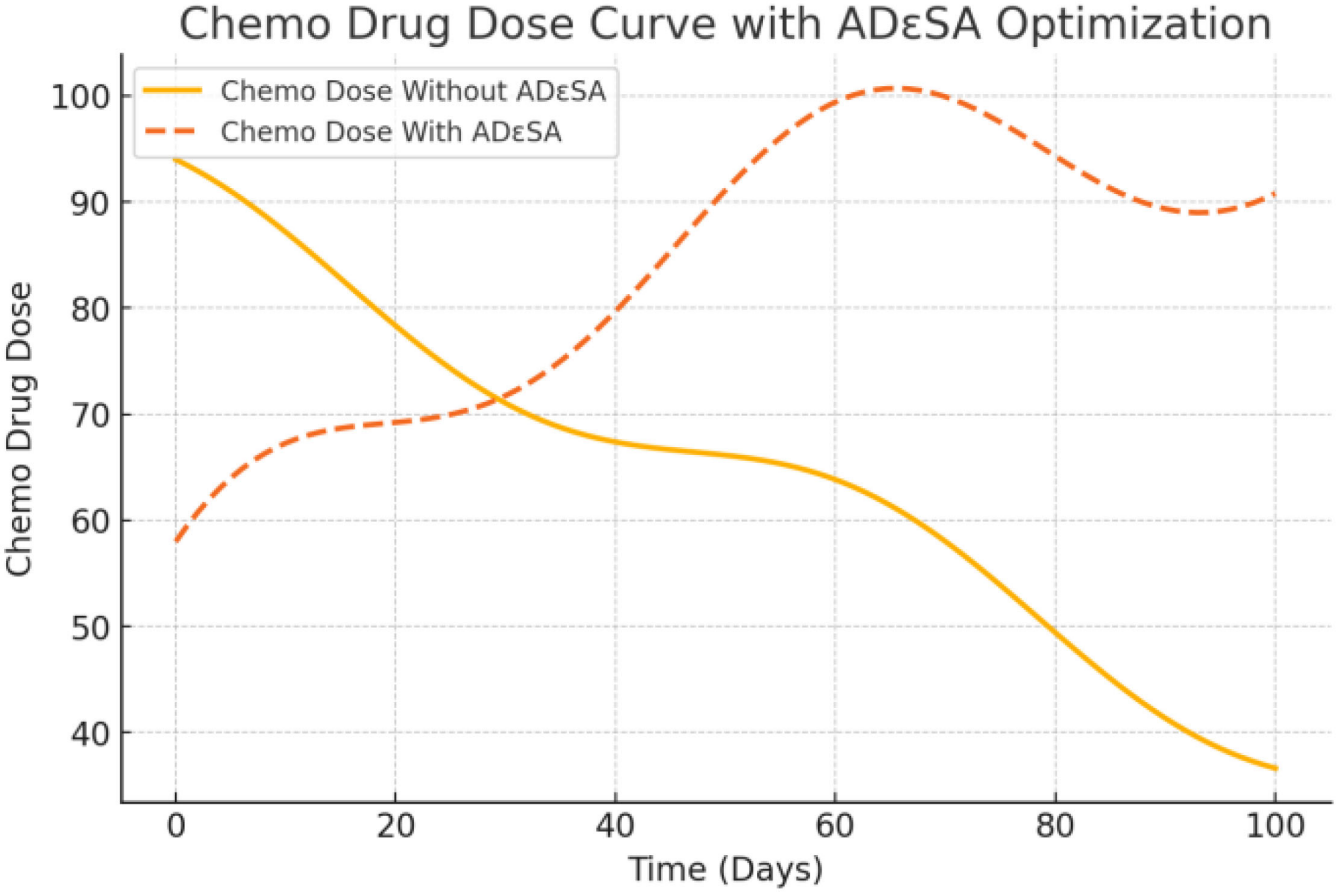
Dosage of chemo drugs’ dose under therapeutic strategies.

**Figure 14** shows that no ADϵSA dosage was used, which showed a simple linear decrease over time. It was not dynamically adjusted according to the therapeutic effect and was under-optimized, which may result in less-than-optimal drug effects. With ADϵSA, the dosage was optimized to show a nonlinear change, with a medium dosage at the beginning to activate the immune system and a gradual increase in the middle and late stages to address excessive immune stimulation. The fluctuation of the curve reflects the dynamic response to the individual state and the flexibility of ADϵSA under multi-stage control. ADϵSA can control the dosage more rationally in the optimal allocation of immune drugs, which improves the initial therapeutic effect and reduces the side effects in the later stage.

**Figure 15:**
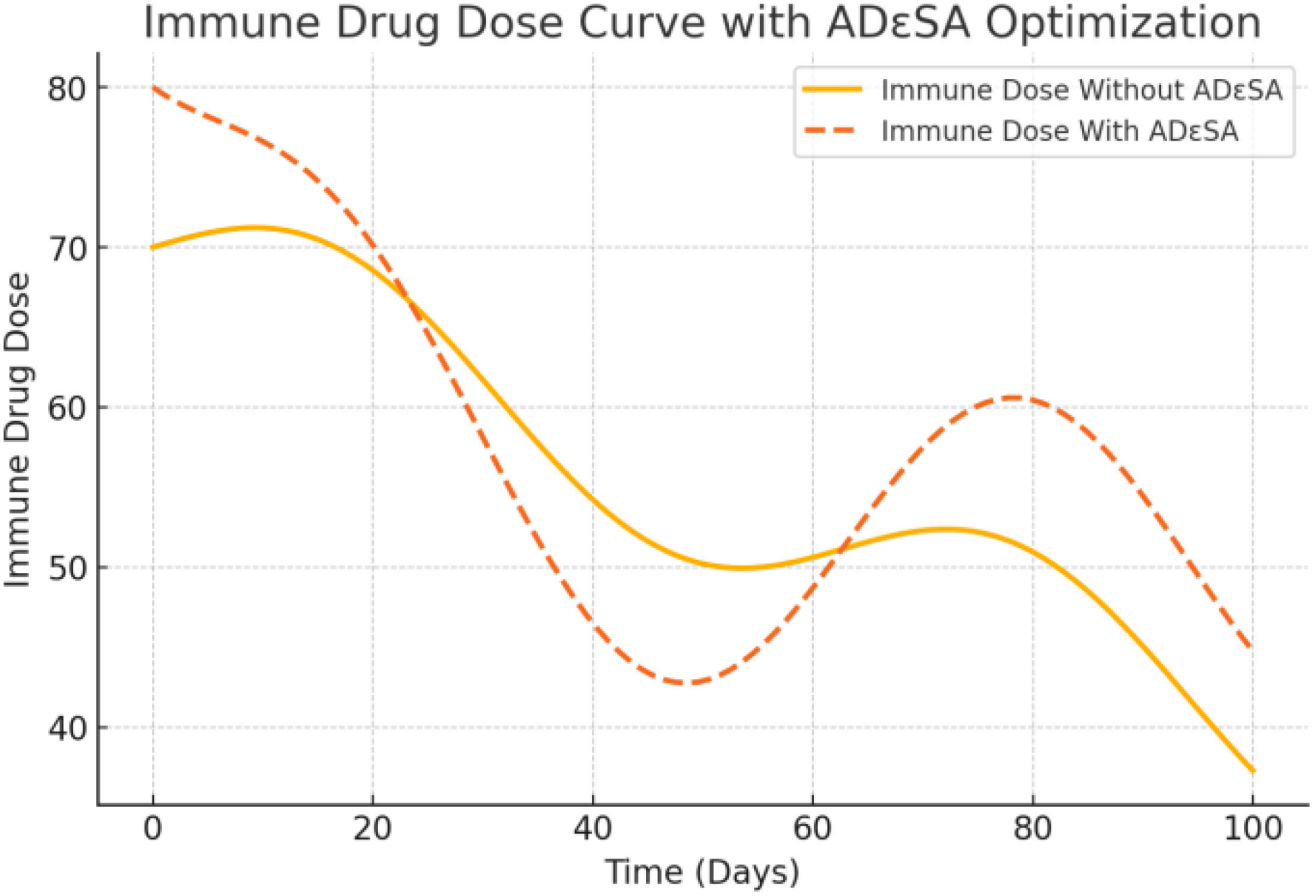
Dosage of immune drugs’ dose under therapeutic strategies.

**Figure 15** shows that without ADϵSA, chemotherapeutic drug dosages simply decrease linearly, without taking into account individual tolerance to chemotherapy or changes in tumor cell response. This may lead to under- or over-dosage, reducing the therapeutic efficacy or increasing the toxic side effects. The optimized curves show that chemotherapeutic agents are used in higher dosages at the initial stage to rapidly inhibit tumor cell growth, gradually decrease in the middle stage to reduce the toxic burden, and remain stable in the later stage. The fluctuating portion reflects the adaptive adjustment of ADϵSA to drug tolerance and tumor response. The optimized chemotherapy dosage of ADϵSA is more in line with personalized treatment needs and is able to balance the intensity of treatment with toxicity and side effects. The output dosage profiles align with adaptive oncology principles: high initial chemotherapy dose reduces tumor burden, followed by a taper to mitigate toxicity. Immunotherapy dosing exhibits a controlled decline, supporting prolonged immune engagement. Such patterns may support clinical decision-making in dose scheduling.

In summary, the advantage of ADϵSA is the rational allocation of drug dosage, and the dosage of immune drugs and chemotherapeutic drugs is dynamically adjusted over time, which not only improves the therapeutic effect but also avoids unnecessary wastage or toxic side effects. At the same time, with multi-stage optimization, the period is gradually reduced to achieve a balance between treatment and toxicity, and with adaptive adjustment, the fluctuation in the dosage curve reflects the real-time adjustment of individual therapeutic response, indicating that ADϵSA can dynamically adapt to the changes in a patient’s condition. It can be combined with global and local; the optimization process can not only jump out of the local optimal solution but also refine the dosage adjustment in each treatment stage, which can comprehensively improve the efficiency of drug use.

## 6 Conclusions

Through the optimization of ADϵSA, the dosage of immune drugs and chemotherapeutic drugs can be allocated more scientifically and rationally. Compared with traditional methods, ADϵSA has faster convergence speed, stronger global search capability, and better optimization effect, which significantly improve the therapeutic effect and reduce the toxic side effects. This treatment strategy based on intelligent optimization algorithm is not only important in anti-tumor treatment but also can be extended to other personalized treatment scenarios, providing new technical support for precision medicine. In the future, in immunotherapy, intelligent optimization algorithms can be used to design and optimize drug combination therapies, match patients and clinical therapies using biomarkers and electronic health records, and personalize cancer treatment. In chemotherapy, intelligent optimization algorithms can be used to accelerate drug discovery and design, use game theory and neural networks to regulate experimental treatment dosage, reduce drug resistance and toxicity, and improve treatment efficacy. For the algorithmic level, the global search mechanism can be enhanced, such as introducing hybrid optimization algorithms (e.g., genetic algorithms combined with local search) or adaptive search strategies. For complex functions, the accuracy control and search capability at the later stage of the algorithm can be improved. On high-density multi-peak functions, perturbation mechanisms or dynamic search range strategies can be used to enhance the comprehensiveness of the results. Future work will explore the integration of ADϵSA into real-time adaptive control frameworks using rolling-horizon optimization and live clinical feedback to dynamically adjust treatment regimens in response to patient response data.

## Data availability of statement

The original contributions presented in the study are included in the article/supplementary material. Further inquiries can be directed to the corresponding author.
