# Correction: Adaptive dynamic ϵ-simulated annealing algorithm for tumor immunotherapy

**DOI:** 10.3389/fimmu.2025.1650988

**Published:** 2025-08-25

**Authors:** Xiaoyan Sun, Ying Jiang

**Affiliations:** ^1^ Department of Gynaecology, The People’s Hospital of Liaoning Province, Shenyang, China; ^2^ Department of Gynaecology, Laoning Cancer Hospital and Institute, Shenyang, China

**Keywords:** tumor immunotherapy (TIT), adaptive dynamic ϵ-simulated annealing algorithm (ADϵSA), chemotherapy and immunotherapy, therapeutic strategies, tumor cells

In the published article, there was an error in the affiliation(s). Affiliation 2, “Department of Gynaecology, Laoning Cancer Hospital and Institute, Shenyang, China”, was erroneously omitted from the author Xiaoyan Sun. It should be: “Xiaoyan Sun^1,2^”.The original article has been updated.

